# Acute Cholangitis Caused by Migration of a Pancreatic Stent Into a Biliary Stent

**DOI:** 10.1002/jhbp.12155

**Published:** 2025-05-09

**Authors:** Ryo Jimbo, Toshifumi Sato, Shuji Terai

**Affiliations:** ^1^ Division of Gastroenterology Saiseikai Niigata Hospital Niigata Japan; ^2^ Division of Gastroenterology and Hepatology Graduate School of Medical and Dental Sciences, Niigata University Niigata Japan

**Keywords:** bile ducts, cholangitis, jaundice, pancreatic ducts, stent

## Abstract

Jimbo and colleagues report a rare case of acute cholangitis caused by migration of a pancreatic stent into a biliary stent, despite appropriate placement. The pancreatic stent obstructed the biliary stent, underscoring the need for careful placement of biliary stents to prevent migration‐related complications following endoscopic procedures.

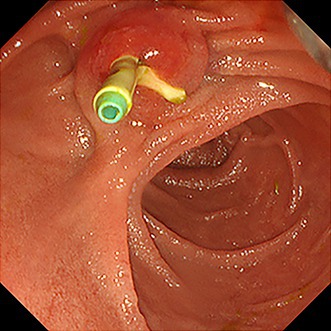

A 74‐year‐old man with obstructive jaundice due to ampullary carcinoma received endoscopic retrograde cholangiopancreatography (ERCP), and a temporary pancreatic stent (Geenen Pancreatic Stent Set with No Internal Flap, 5 Fr, 3 cm; Cook Medical, Bloomington, IN, USA) and a biliary plastic stent (Advanix J, Straight, 7 Fr, 7 cm; Boston Scientific, Marlborough, MA, USA) were placed to relieve obstructive jaundice. The pancreatic stent was placed to prevent post‐ERCP pancreatitis (PEP) because bile duct cannulation is relatively time‐consuming, and endoscopic sphincterotomy was not performed. The patient was discharged 4 days after ERCP. Three days later, however, he was readmitted with fever and jaundice. Repeat ERCP revealed that the pancreatic stent had migrated into the biliary stent (Figure 1). Both stents were grasped with forceps and pulled out without resistance. An endoscopic nasobiliary drainage tube was inserted, and the patient's cholangitis improved. In this case, the pancreatic stent migrated into the biliary stent, resulting in biliary stent obstruction. Retrospective review of the endoscopic image taken during ERCP showed that both stents were placed in the appropriate position (Figure 2a), but the day after stent placement, abdominal radiography revealed that the pancreatic stent had migrated into the biliary stent (Figure 2b). The pancreatic stent appears to have migrated through the flap of the biliary stent (Figure 2c). This phenomenon may have occurred because the biliary stent was not inserted far enough to the distal flap, creating a space into which the pancreatic stent could migrate. Although pancreatic stents are effective for preventing PEP [1, 2], endoscopists should be aware that pancreatic stents can migrate into biliary stents owing to their close proximity and different diameters, and the biliary stent should be carefully placed so that the distal flap is just adjacent to the papilla of Vater.

**FIGURE 1:**
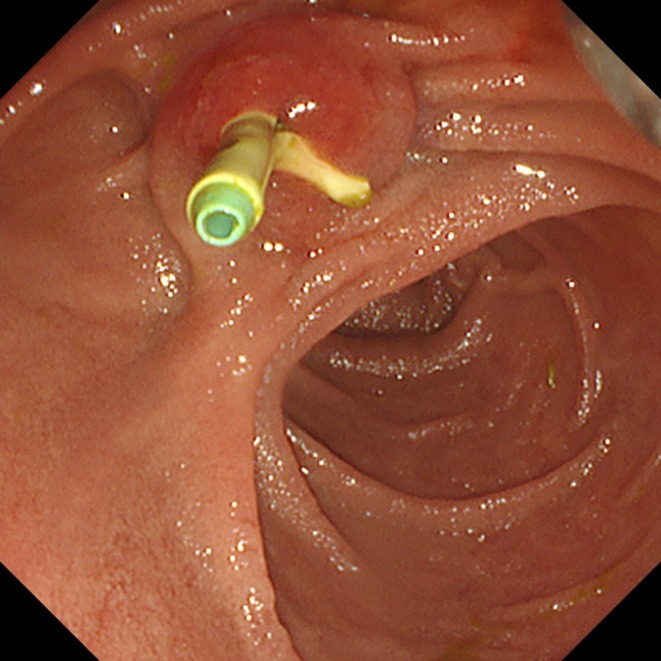
Endoscopic retrograde cholangiopancreatography (ERCP) photograph showing migration of the distal end of the pancreatic stent into the biliary stent.

**FIGURE 2:**
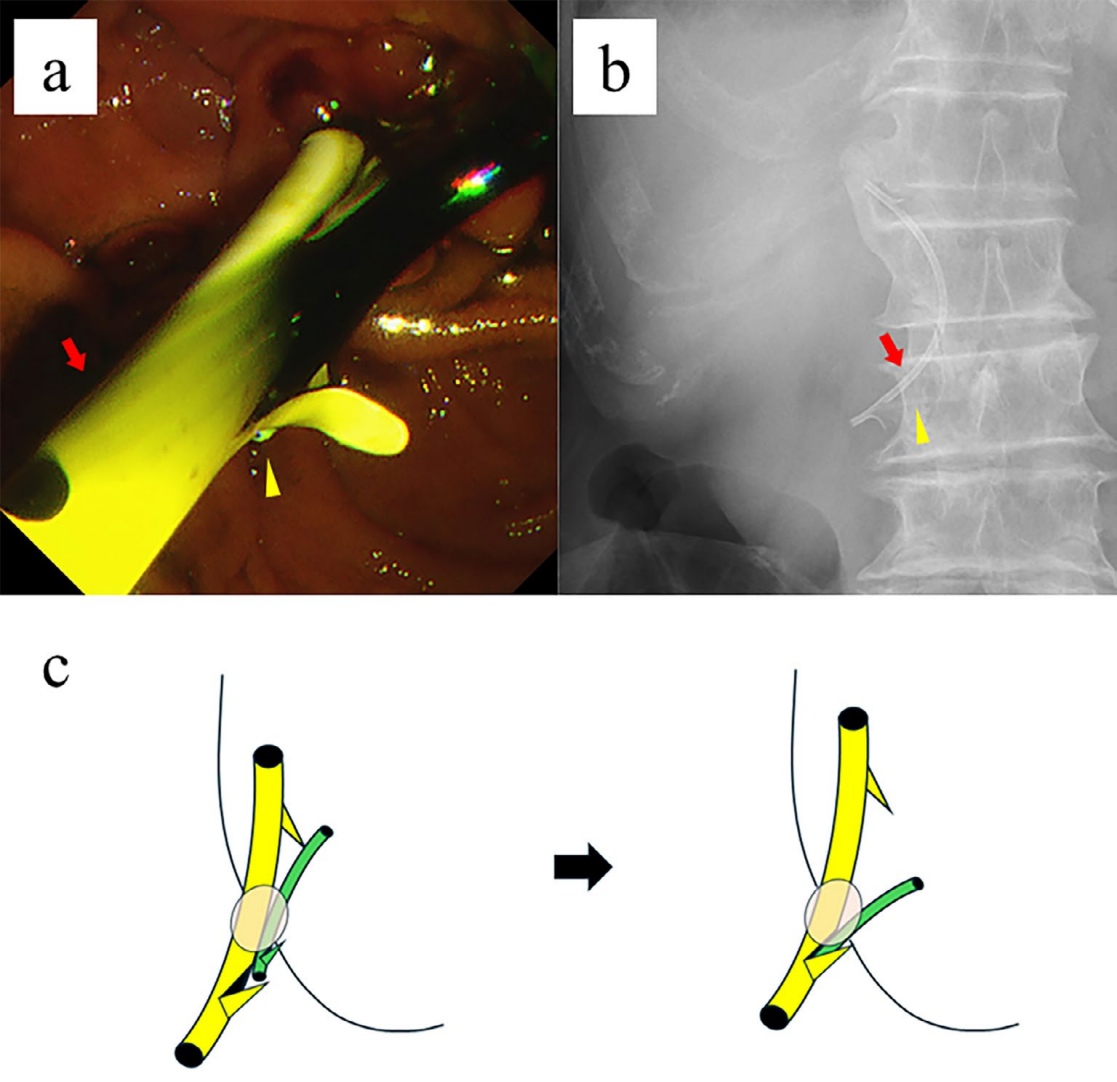
(a) Endoscopic image taken during endoscopic retrograde cholangiopancreatography (ERCP) showing the original position of the stents. The yellow arrowhead shows the distal end of the pancreatic stent and the red arrow shows the biliary stent. (b) Abdominal radiography performed the day after stent placement showing migration of the pancreatic stent into the biliary stent. The yellow arrowhead shows the pancreatic stent and the red arrow shows the biliary stent. (c) Pictorial representation of the migration of the pancreatic stent into the biliary stent through a flap in the biliary stent.

## Conflicts of Interest

The authors declare no conflicts of interest.

## Data Availability

Data sharing not applicable to this article as no datasets were generated or analysed during the current study.
